# UAV Trajectory Planning for Radar Deployment Assessment

**DOI:** 10.3390/s26216932

**Published:** 2026-11-02

**Authors:** Jan M. Kelner, Jan Łakomy, Piotr Marczak, Robert Przybycień, Wojciech Stecz, Mariusz Woźniak

**Affiliations:** 1 Institute of Communications Systems, Faculty of Electronics, Military University of Technology, Gen. Sylwestra Kaliskiego 2, 00-908 Warsaw, Poland; 2 PIT-RADWAR, Poligonowa 30, 04-051 Warsaw, Poland; jan.lakomy@pitradwar.com (J.Ł.); piotr.marczak@pitradwar.com (P.M.); robert.przybycien@pitradwar.com (R.P.); mariusz.wozniak@pitradwar.com (M.W.); 3 Faculty of Mathematics and Information Science, Warsaw University of Technology, Koszykowa 75, 00-662 Warsaw, Poland; 4 Institute of Computer Sciences, Cardinal Stefan Wyszyński University, Wóycickiego 21, 01-938 Warsaw, Poland

**Keywords:** trajectory planning, Parallel Quick Rapidly-exploring Random Tree Star (PQRRT*), Rapidly-exploring Random Tree (RRT), Fast Marching Method (FMM), Fast Marching Square (FM^2^), Covariance Matrix Adaptation Evolution Strategy (CMA-ES)

## Abstract

**Highlights:**

**What are the main findings?**

- The proposed framework employs Parallel Quick Rapidly-exploring Random Tree* (PQRRT*), Fast Marching Method (FMM), and Covariance Matrix Adaptation Evolution Strategy (CMA-ES) to generate unmanned aerial vehicle (UAV) trajectories that minimize the probability of detection in radar-covered areas.
- PQRRT* enables parallel trajectory planning on large two- (2D) grids while achieving computational efficiency comparable to FM method and significantly outperforming evolutionary methods.

**What are the implication of the main finding?**

- The generated UAV trajectories reveal potential gaps in radar coverage and provide an effective means of assessing radar deployment against low-altitude aerial threats.
- The proposed approach can support radar-coverage assessment by enabling rapid verification and adjustment of radar locations in large-scale, real-world scenarios.

**Abstract:**

Optimal radar deployment is an important element in designing surveillance and monitoring systems for protected areas, particularly in applications involving low-altitude unmanned aerial vehicles (UAVs). Assessing a given radar configuration requires identifying potential UAV trajectories that exploit regions of reduced radar coverage and may therefore result in delayed detection. This paper presents a trajectory-planning framework for radar deployment assessment based on three complementary approaches: Parallel Quick Rapidly-exploring Random Tree* (PQRRT*), Fast Marching Method (FMM), and Covariance Matrix Adaptation Evolution Strategy (CMA-ES). The radar coverage area is represented by a discretized spatial grid in which each cell is characterized by the probability of detecting a UAV with a specified radar cross-section at a given flight altitude. The trajectory-planning problem is formulated to identify routes that minimize the cumulative probability of detection before reaching a predefined target region. The considered algorithms are adapted to operate in such continuous radar-coverage fields rather than only in environments containing discrete obstacles. In addition, the proposed PQRRT* algorithm employs parallel computation and non-blocking data structures, enabling efficient trajectory generation over large-scale areas represented by grids containing millions of cells. Numerical experiments demonstrate that PQRRT* and FMM efficiently generate multiple UAV trajectories in large-scale radar-coverage scenarios, whereas CMA-ES requires substantially higher computational effort for more complex optimization settings. The generated trajectories enable the identification of potential coverage gaps and provide quantitative information for assessing alternative radar configurations. The results demonstrate that the proposed framework can support radar-coverage assessment and assist in improving radar deployment for UAV monitoring and protection of critical areas.

## 1. Introduction

Swarms of unmanned aerial vehicles (UAVs) are becoming increasingly common in a wide range of applications requiring coordinated operation of multiple aerial platforms. At the same time, the capabilities of drone detection systems, including optronic sensors and passive and active radars, are advancing significantly.

Despite advances in UAV detection and counter-UAV systems, the increasing availability, relatively low cost, and coordinated operation of multiple UAVs continue to pose significant challenges to the effective monitoring and protection of critical areas. Effective monitoring and protection against UAV swarms is therefore becoming increasingly important. Systems designed to counter airborne threats, including UAVs, generally comprise three functional stages: detection, threat classification and assessment, and threat response.

However, because the cost ratio of UAVs to detection sensors can be highly unfavorable, there is a need to develop methods that support efficient sensor deployment planning and improve the coverage of protected areas. The problem of limited radar coverage, for both active [1,2] and passive [3,4] radar systems, requires an a priori assessment of potential deployment locations and the corresponding radar coverage at a given altitude and direction for an object with a specified radar cross-section (RCS). A common approach to planning the deployment of a given number of radars is to discretize the deployment area into a grid of squares (sometimes hexagons) to estimate approximate coverage. Most research on radar deployment and coverage typically focuses on two-dimensional (2D) areas, which represents a simplification but can be used to model UAV operation at a relatively constant flight altitude.

For a predefined radar deployment and the resulting coverage field, potential UAV flight trajectories should therefore be analyzed to identify approach paths associated with delayed detection. This article presents a method to support the assessment of radar placement (or the adjustment of an existing configuration). The method enables the identification of areas where the probability of detecting UAVs or other airborne objects may be reduced. It is based on determining potential attack trajectories toward a target protected by radars. Trajectory-planning algorithms seek paths that allow for a stealthy approach to the target, ensuring that the detection of the threat occurs as late as possible.

Three methods for generating UAV flight trajectories are presented and subsequently used to assess adjustments of radar positions aimed at increasing the probability of detecting incoming aerial objects. The first algorithm, based on Rapidly-exploring Random Trees (RRT), employs the parallel RRT tree expansion method. It is highly efficient and enables the determination of very precise flight trajectories. The second algorithm uses the Fast Marching Method (FMM), allowing for the efficient calculation of multiple paths for UAVs approaching from different directions. The third algorithm is based on the Covariance Matrix Adaptation Evolution Strategy (CMA-ES) method and is comparatively slower than the others.

This work introduces a novel method for verifying the correct deployment of a finite number of radars in a given area. The method achieves this by generating representative UAV trajectories using FMM, RRT, and CMA-ES algorithms. To facilitate this, a carefully designed objective function, combined with a modified approach for selecting the UAV’s next flight point, enables effective application of the algorithm in route planning within environments containing non-discrete obstacles. In trajectory planning, the presence of a radar field does not necessarily prevent the UAV from entering that area. Furthermore, the Parallel Quick RRT* (PQRRT*) algorithm employs parallel computation and non-blocking data structures to support efficient trajectory generation in large-scale search spaces.

The novelties of our approach are as follows:

1. Trajectory planning is performed in 2D on square grids composed of millions of points.
2. Unlike commonly used RRT algorithms, this algorithm considers not only discrete obstacles to be avoided, but also the radar field coverage, which the drone may fly into if no other flight path is available.
3. The PQRRT* algorithm employs parallel computation and non-blocking data structures to support efficient trajectory generation in large-scale search spaces.

**Figure 1:**
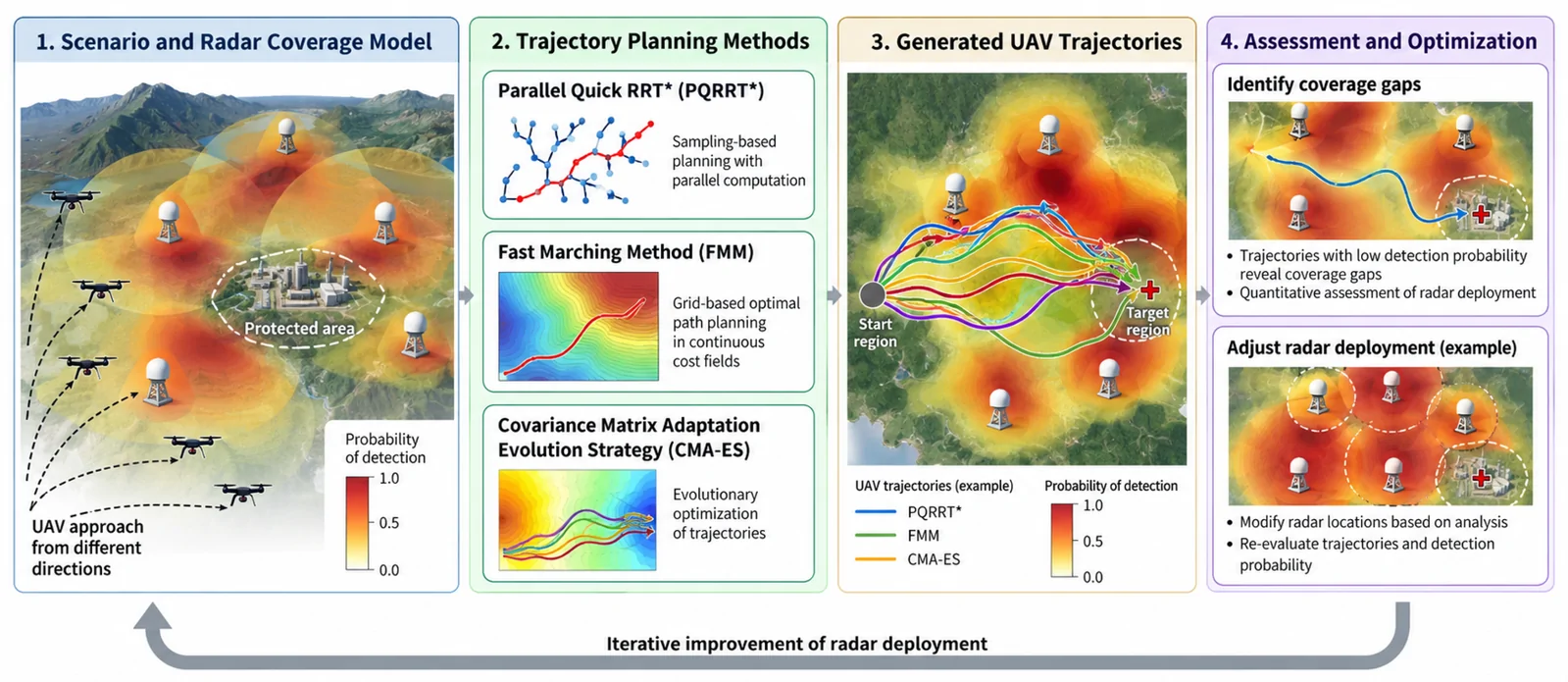
Overall concept of the proposed UAV trajectory-planning framework for radar deployment assessment. Radar coverage is represented by a spatially varying probability of UAV detection. PQRRT*, FMM, and CMA-ES are used to generate representative low-detection-probability trajectories, which reveal potential coverage gaps and support the assessment and iterative adjustment of radar locations.

The overall concept of the proposed radar-deployment assessment framework is illustrated in Figure 1. The procedure starts with a spatial representation of radar coverage expressed in terms of UAV detection probability. PQRRT*, FMM, and CMA-ES are then used to generate representative UAV trajectories associated with reduced detection probability. The resulting trajectories are analyzed to identify potential coverage gaps and to support the assessment and adjustment of radar deployment. After modifying radar locations, the trajectory-planning process can be repeated to evaluate the resulting improvement in radar coverage.

The remaining part of the paper is organized as follows. Section 2 reviews important studies in this area. Section 3 describes the algorithms used to generate UAV flight trajectories for radar-coverage assessment. Section 4 presents selected experimental results from real-world areas spanning several thousand square kilometers, with grid sizes exceeding $10^{6}$ elements (1000 × 1000 grids). The performance of the individual algorithms is also compared. Finally, Section 5 summarizes our results.

## 2. Related Works

Trajectory planning is a key area of research in UAVs. Among various trajectory planning algorithms, the RRT algorithm demonstrates strong search capabilities in both the solution space and random sampling. It also shows significantly greater potential for generating optimal trajectories. For a comprehensive overview of recent advances in RRT algorithms, the reader is referred to the work of [5], which provides an overview of the most important research on RRT algorithms in recent years.

Regarding research on RRT-based trajectory planning algorithms, several modifications have been proposed to improve performance. However, the RRT algorithm still exhibits several limitations in terms of trajectory planning accuracy, indicating that further optimization is required for platform path planning. In the paper [6], a path correction method was used to improve the trajectory found by the RRT.

The RRT algorithms presented in this paper are based on our previous research on trajectory determination using RRT [7,8]. In the study by Kelner et al. [8], a method was proposed for determining the flight trajectories of several drones flying at a distance from each other while maintaining communication links. The paper by Burzynski et al. [7] provides a detailed description of an algorithm for determining the flight trajectories of several drones in an environment with obstacles. In this case, the drones were required to fly in proximity to each other while maintaining a minimum safe separation distance. This type of task is a classic multi-UAV trajectory-planning problem, where coordinated flight at specific relative distances is essential.

In this paper, the RRT algorithm is applied differently than the approaches of Burzynski [7] and Kelner [8]. The objective of the trajectory-planning task is to generate paths that originate from a common initial region and represent multiple approach directions within the radar-covered area. Each trajectory is subsequently optimized with respect to the spatially varying probability of radar detection, enabling potential coverage gaps to be identified. This study employs and modifies a parallel implementation of the RRT algorithm described by Ichnowski et al. [9].

Alvarez et al. [10] present a trajectory planning algorithm for 3D robot formations based on the standard Fast Marching Square (FM^2^) path planning method. The FM^2^ algorithm is a motion planning method that generates smooth, suboptimal paths for autonomous systems. It also accounts for dynamic changes in obstacle positions. It operates by constructing a “velocity map” in which the wave-propagation speed varies with the environment’s characteristics, and then applying the fast marching algorithm to determine the optimal path. The method has been extended to support leader-follower robot formation planning—the classic, most widely used formation approach for drone swarms. In this framework, the reference positions of the following platforms are defined using equations that depend on the leader’s position. In this respect, the algorithm is based on the assumptions used by Kelner [8]. The algorithm also accounts for obstacles and operates fully in 3D.

The paper by Garrido et al. [11] presents a method for planning flight paths for UAV formations in 3D environments. The paths were designed for search-and-rescue missions, where the generated trajectories had to ensure coverage of the entire designated search area. The proposed approach uses the FM^2^ algorithm for path planning.

The paper by Gonzales et al. [12] presents an example of 3D UAV flight path planning that accounts for the vehicle’s maneuverability. Dubin curves were used to model the realistic target behavior. The model was developed for an autonomous fixed-wing aircraft. Some aircraft models can be found in [13,14].

In this paper, we build on the FMM method developed by Furtney [15], which is available on GitHub. We employ a modified version of the trajectory-planning algorithm in areas covered by radar systems. Obstacles are modeled as continuous rather than discrete, and each platform must be able to move within the area where radars track it.

In the work of Dreo et al. [16], the FMM algorithm is applied for radar deployment planning. Trajectory computations are performed using a variant of the FMM algorithm that minimizes the UAV’s flight energy consumption.

A related perspective on trajectory planning based on spatially varying risk-related costs is presented by Liu et al. [17], who investigated trajectory planning for traffic safety with dynamic ethical risk adjustment. Although their application differs from radar-deployment assessment, the study illustrates how risk-related factors can be incorporated into trajectory-planning objectives. In contrast, the present work focuses on radar detection-probability fields and the computational efficiency of algorithms for generating representative UAV trajectories in large-scale spatial grids.

Our paper fills a gap in the literature regarding heuristic algorithms such as RRT. Conventional RRT-based planning methods typically represent obstacles as discrete forbidden regions and do not directly account for spatially varying continuous cost fields. This study demonstrates how an RRT-based approach can be adapted to trajectory planning in radar-coverage fields represented by continuous detection-probability values.

## 3. Materials and Methods

Following an overview of the physical principles underlying radar-based object detection, this section describes selected algorithms for determining drone flight trajectories, designed for parallel computation. Parallelizing the solution-space search process is particularly important for trajectory planning tasks involving areas modeled with a large number of components.

### 3.1. Radar-Based Object Detection

In radar system analysis, the radar range equation provides a fundamental relationship between transmitted power, antenna parameters, target RCS, propagation losses, and received signal power. For a conventional monostatic radar, this relationship is expressed as [18]:

(1) $$P_{r}=\frac{P_{t}G^{2}\lambda^{2}\sigma }{{\left(4\pi \right)}^{3}R^{4}L},$$

where $P_{r}$ is the received power, $P_{t}$ is the transmitted power, *G* is the antenna gain, λ is the wavelength, σ is the target RCS, *R* is the range to the target, and *L* represents system and propagation losses. This model represents the key factors influencing radar detection capabilities and serves as a standard analytical approach in radar research and system design.

It is important to note that the radar cross section of a target depends on several factors, including the radar frequency, polarization, aspect angle, and target geometry. For complex targets, such as fixed-wing aircraft or large UAVs, the RCS may exhibit significant fluctuations (even up to 40 dB) due to changes in viewing geometry and interference among contributions from individual scattering centers. Therefore, a fixed-RCS model cannot fully reproduce dynamic fluctuations in the detection range. Accurate modeling of these complex phenomena has been extensively investigated in the literature [18]. However, since this study focuses on detection probability only, assuming a constant RCS is considered a reasonable simplification.

Another set of factors that should be taken into account in accurate radar modeling relates to atmospheric refraction and multipath propagation, particularly reflections from the Earth’s surface, which can cause constructive or destructive interference between signals arriving via different propagation paths. This results in range- and altitude-dependent fluctuations in received power, commonly referred to as multipath fading. Such effects are not represented by the adopted free-space propagation model and may lead to local variations in detection performance that are absent from the simulated coverage.

Comprehensive radar modeling that accounts for propagation effects (including multipath phenomena), antenna radiation patterns, and system-level interactions is a complex process that lies beyond the scope of this study. Instead, the analysis concentrates on estimating detection probability under a set of simplifying assumptions.

The operational area was assumed to be divided into equal-sized squares, forming a grid in which each square represents a portion of the area. For each square, a function was defined to describe the quality of radar signal coverage at a specific scale, which determines the probability of a single object detection. In this paper, we assume that the coverage values lie in the range [0, 1], where 1 indicates that the radar detects an object of a given type within the area represented by the square, and 0 indicates that the object will not be detected in that area.

We model the probability using the following formula:

(2) $$P^{D}=1-P_{\gamma }\left(\frac{P_{\gamma }^{-1}\left(1-P_{FA};1\right)}{SNR+1};1\right),$$

where the false detection probability $P_{FA}$ is taken as $10^{-6}$. $P_{\gamma }$ is the incomplete gamma function given by:

(3) $$P^{\gamma }\left(x;\alpha \right)=\frac{1}{\Gamma (\alpha )}\int_{0}^{x}t^{\alpha -1}e^{-t}dt.$$

Based on the radar Equation (1), the signal-to-noise ratio (SNR) can be expressed as the ratio of the received signal power $P_{r}$ to the thermal noise power $P_{n}$, yielding:

(4) $$SNR=A\frac{\sigma }{R^{4}},$$

where *A*, according to Equation (1), depends only on the radar model and atmospheric conditions, which are assumed to be constant. Assuming that each individual radar detection (2) $P_{i}$ occurs independently, the detection probability (cost function) of the entire surveyed route can be defined as:

(5) $$P_{1\ldots N}=1-\Pi_{i=1}^{N}\left(1-P_{i}\right)$$

(6) $$1-P_{1\ldots N}=\Pi_{i=1}^{N}\left(1-P_{i}\right)$$

(7) $$\log\left(1-P_{1\ldots N}\right)=\sum_{i=1}^{N}\log\left(1-P_{i}\right)$$

Accordingly, minimizing the cumulative detection probability is equivalent to minimizing the negative logarithm of the probability of remaining undetected along the trajectory, as obtained from Equation (7). For radar-deployment assessment, it is particularly important to identify trajectories along which detection may be delayed until the UAV reaches a predefined target region. Therefore, a perimeter is defined around the target region, and the path cost is calculated as the negative logarithm of the probability of remaining undetected before reaching this perimeter.

The cumulative detection-probability formulation assumes statistical independence between the individual detection events represented by $P_{i}$. In practice, successive detection events may be correlated due to radar scanning characteristics, target motion, and propagation conditions. Therefore, the resulting cumulative detection probabilities should be interpreted as model-based estimates rather than exact probabilities of detection under all operating conditions.

Due to significant differences between the presented algorithms, the notations used in the pseudocodes apply to the algorithm described in a given section only.

### 3.2. Rapidly-Exploring Random Trees

RRT algorithms are effective for planning drone flight trajectories [7,8]. The popularity of RRT has led to extensive research into its application for trajectory calculation in unmanned platforms, including UAVs. Numerous RRT variants exist, including those that support parallel trajectory computation (e.g., [19,20]).

The primary algorithm tested in this work for generating drone flight trajectories over large areas modeled as square grids is PQRRT*. It is based on the Quick-RRT* method proposed by Jeong [21], with the parallel version initially introduced for standard RRT* by Ichnowski [9].

#### 3.2.1. Parallel Quick RRT*

To enable efficient parallelization of the algorithm, the algorithm employs three data structures that differ from those used in the standard version of Quick-RRT*:

- Non-blocking k-d tree (denoted *T*):
  It works like a standard k-d tree, but allows multiple threads to access it concurrently without blocking. The algorithm uses it to efficiently search for the next waypoints in the UAV’s flight path.
- Vertex structure:
  A structure stores its location and incident edge, which connect it to its parent. In practice, a vertex represents a point where the UAV changes direction. For any point *x* in a search space, the node structure assigned to this point is denoted as node(x).
- Edge structure:
  In the presented algorithm, an edge stores the vertex it connects to, the cost of the path from the starting vertex, and its children, represented as edge structures. The edge links a specific vertex to the rest of the tree. Each edge uniquely defines a single path from the starting vertex (the tree’s root) to its incident vertex. For any point *x* in a search space, the edge structure assigned to this point is denoted as edge(x).

The PQRRT* algorithm determines a path to the target by minimizing a trajectory cost function derived from the spatially varying radar detection probabilities, as described in Section 3.1.

The algorithm initiates by inserting a starting node $x_{begin}$ into the tree *T*, followed by the launch of a defined number of execution threads (see Algorithm 1).

Algorithm 1 can be executed across multiple threads. In this case, the solution space can be divided into disjoint subspaces according to a chosen scheme, with each subspace assigned to a single thread. Here a trivial space partitioning was adopted – each thread was assigned a vertical slice of equal width but other, more sophisticated schemes can be used.

Each thread in the loop attempts to add a new vertex until the size of the tree *T* (denoted |T|) reaches the maximum value *n*. First, the function sample() generates a vertex $x_{sample}$ representing a random point in the given region, which serves as a potential point of the tree *T*. Subsequently, the function nearest() finds the vertex $x_{nearest}$ in the tree *T* that is closest to $x_{sample}$. Then, the function steer creates a new vertex $x_{new}$ by moving $x_{sample}$ towards $x_{nearest}$, ensuring that the distance between $x_{nearest}$ and $x_{new}$ is less than or equal to the maximum step length $l_{max}$. The lower the value of the parameter $l_{max}$, the more iterations are required to reach a desirable solution, however, the found paths are smoother, consisting of more segments. Here a value of 200 m was chosen as a good tradeoff between the two.
**Algorithm 1** Single PQRRT* thread**Require:** $x_{begin}$, $x_{end}$, single step length $l_{max}$, maximum number of vertices *n*, maximum depth $d_{max}$, lock-free k-d tree *T* **Ensure:** path *p* from $x_{begin}$ to $x_{end}$
  1:**while** |T|<n **do**  2:      $x_{sample}\leftarrow sample\left(\right)$  3:      $x_{nearest}\leftarrow nearest\left(T,x_{sample}\right)$  4:      $x_{new}\leftarrow steer\left(x_{nearest},x_{sample},l_{max}\right)$  5:      **if** $valid(x_{nearest},x_{new})$ **then**  6:         $\chi_{close}\leftarrow closeTo(T,x_{new},r(|T|,l_{max}))$  7:         $\chi_{ancestors}\leftarrow ancestors\left(\chi_{close},d_{max}\right)$  8:         $c_{min}\leftarrow pathCost\left(edge\left(x_{nearest}\right)\right)+cost\left(x_{nearest},x_{new}\right)$  9:         $x_{best}\leftarrow x_{nearest}$10:         **for** $x_{close}\in \chi_{close}\cup \chi_{ancestors}$ **do**11:               $c_{close}\leftarrow pathCost\left(edge\left(x_{close}\right)\right)+cost\left(x_{close},x_{new}\right)$12:               **if** $c_{close}<c_{min}\wedge valid\left(x_{close},x_{new}\right)$ **then**13:                  $c_{min}\leftarrow c_{close}$14:                  $x_{best}\leftarrow x_{close}$15:               **end if**16:         **end for**17:         $e_{parent}\leftarrow edge\left(x_{best}\right)$18:         $e_{new}\leftarrow \left(e_{parent},x_{new},c_{min}\right)$19:         $edge\left(x_{new}\right)\leftarrow e_{new}$20:         $updateBestPath(e_{new},x_{target},l_{max})$21:         $addChild(e_{parent},e_{new})$22:         $treeInsert(T,x_{new})$23:         **if** $expired(e_{parent})$ **then**24:               $update(e_{parent},node\left(e_{parent}\right))$25:         **end if**26:         **for** $x_{close}\in \chi_{close}$ **do**27:               $rewire(x_{new},x_{close})$28:         **end for**29:      **end if**30:**end while**

Additionally, the path between vertices must be free of obstacles.

Then the valid() function checks whether the vertices $x_{nearest}$ and $x_{new}$ can be correctly connected; if not, the current loop iteration is terminated.

In the following lines, the algorithm attempts to find the best parent for vertex $x_{new}$—the closeTo() function identifies, using the tree *T*, the set $\chi_{close}$ of all vertices that are closer to $x_{new}$ than the distance computed by the function $r(size,l_{max})$, given by $\min\left\{l_{max},\gamma \;\cdot \;\sqrt{\frac{\log(size)}{size}}\right\}$, where γ is a constant from the function *r* shown in [7]. Next, the ancestors() function identifies the set of ancestors, of degree at most $d_{max}$, of vertices from the set $\chi_{close}$. Increasing the value of the parameter $d_{max}$ decreases the number of iterations required to find a desirable path, at the cost of increasing the runtime of a single iteration. In this case a value of 2 was chosen as it was recommended in [21] for complex environments.

The algorithm then iterates over all vertices $\chi_{close}\cup \chi_{ancestors}$ searching for one that can be properly connected to $x_{new}$, and if chosen as the parent of $x_{new}$, it minimizes the path cost to $x_{new}$.

The edge() function is used to access the current edge of a given vertex, and the cost() function is used to calculate the cost of a connection between two vertices, based on both the cover value and the distance between them.

After selecting a parent for $x_{new}$, the algorithm updates the best path found using the updateBestPath() procedure. The algorithm then adds the edge $e_{new}$ (assigned to vertex $x_{new}$) to the list of children of edge $e_{parent}$ (assigned to vertex $x_{best}$). Finally, the vertex $x_{new}$ is added to the tree *T*, enabling efficient vertex search.

Since the path determined by the algorithm and the cost represented by a given edge remain constant, to determine the path leading to the RRT tree vertex with the lowest cost, the current edge is replaced with a new one. This replacement may cause some edges to become “expired”, meaning they no longer represent valid edges for their vertices. The expired(e) function detects such edges. The update(e,x) function corrects the corresponding connections.

The child edges are stored in a thread-safe, non-blocking list that provides operations to add an element (addChild), remove the first element (removeFirstChild), and remove the selected element (removeChild).

The treeInsert operation, presented in Algorithm 2, traverses the tree to find a suitable, free slot to insert a new vertex $n_{new}$. It then attempts to insert it using the CAS operation, which, for the currently examined vertex $n_{current}$, checks whether $n_{current}$ has two other child vertices attached. If not, it attaches it to the appropriate side of the tree *T*. If the replacement is successful, the procedure terminates; otherwise, the search continues from the vertex that occupied the previously selected free slot.
**Algorithm 2** treeInsert**Require:** $T,x_{new}$    $n_{new}\leftarrow \left(x_{new},NULL,NULL\right)$    $n_{current}\leftarrow T.root$    **for** depth←0... **do**    axis←depthmod2    **if** $n_{new}.node.point\left[axis\right]<n_{current}.node.point\left[axis\right]$ **then**      $n_{left}\leftarrow n_{current}.left$      **if** $n_{left}=NULL\wedge CAS\left(n_{current}.left,NULL,n_{new}\right)$ **then return**      **end if**      $n_{current}\leftarrow n_{current}.left$    **else**      $n_{right}\leftarrow n_{current}.right$      **if** $n_{right}=NULL\wedge CAS\left(n_{current}.right,NULL,n_{new}\right)$ **then return**      **end if**      $n_{current}\leftarrow n_{current}.right$    **end if**    **end for**

The next step is to check whether $e_{parent}$ has become “expired”. If it is the case, the algorithm updates the children of $e_{parent}$ by invoking Algorithm 3 for the edge $e_{parent}$ and its assigned vertex (accessed using the node() function).

Finally, the algorithm attempts to rewire the vertices from $\chi_{close}$ through $x_{new}$ using the rewire() procedure, presented in Algorithm 4.
**Algorithm 3** update**Require:** $e_{old},x$  1:$e_{new}\leftarrow edge\left(x\right)$  2:**while** true **do**  3:   $e_{child}\leftarrow removeFirstChild\left(e_{old}\right)$  4:   **if** $e_{child}=NULL$ **then**  5:         **if** $expired(e_{new})$ **then**  6:         $update(e_{new},node\left(e_{new}\right))$  7:         **end if return**  8:   **end if**  9:   **if** $\neg expired(e_{child})$ **then**10:         $x_{child}\leftarrow node\left(e_{child}\right)$11:         $c_{child}\leftarrow pathCost\left(e_{new}\right)+cost\left(e_{new},e_{child}\right)$12:         $e_{newChild}\leftarrow \left(e_{new},x_{child},c_{child}\right)$13:         **if** $swapEdge(x_{child},e_{child},e_{newChild})$ **then**14:         $addChild(e_{new},e_{newChild})$15:         $update(e_{child},e_{newChild})$16:         **end if**17:   **end if**18:**end while**

**Algorithm 4** rewire**Require:** $x_{new}$, $x_{close}$, $d_{max}$  1:**for** $x_{current}\in x_{new}\cup ancestors\left(\left\{x_{new}\right\},d_{max}\right)$ **do**  2:   **if** $\neg valid(x_{current},x_{close}$) **then**  3:       **continue**  4:   **end if**  5:   $e_{parent}\leftarrow edge\left(x_{current}\right)$  6:   $c_{current}\leftarrow pathCost\left(e_{parent}\right)+cost\left(x_{current},x_{close}\right)$  7:   $e_{current}\leftarrow \left(e_{parent},x_{close},c_{current}\right)$  8:   failed←false  9:   **repeat**10:       $e_{old}\leftarrow edge\left(x_{close}\right)$11:       **if** $c_{current}\geq pathCost\left(e_{old}\right)$ **then**12:        failed←true13:       **end if**14:   **until** $\neg failed\wedge swapEdge(x_{close},e_{old},e_{current})$15:   **if** failed **then**16:       **continue**17:   **end if**18:   $addChild(e_{parent},e_{current})$19:   $update(e_{old},e_{current})$20:   **if** $expired(e_{parent})$ **then**21:       $update(e_{parent},edge\left(x_{current}\right))$22:   **end if**23:   $removeChild(parent\left(e_{old}\right),e_{old})$24:**end for**

#### 3.2.2. Update

Edges $e_{old}$ and $e_{new}$ are both connected to the same vertex *x*. If edge $e_{old}$ is replaced in *x* by edge $e_{new}$ (it becomes “obsolete”), then all descendants of $e_{old}$ must also be replaced in their vertices by new edges containing the updated cost and connected to a new path via $e_{new}$.

This is achieved by using the update() procedure, which first assigns the current edge of vertex *x* to the variable $e_{new}$. Subsequently, it removes the edges that are children of edge $e_{old}$ one by one, and for each such “not expired” edge $e_{child}$, it attempts to replace it with a new edge $e_{newChild}$ whose path passes through $e_{new}$, and the cost is the cost associated to the new path. To perform this replacement, the $swapEdge(x_{child},e_{child},e_{newChild})$ function is used. This is a single atomic instruction that first checks whether $edge\left(x_{child}\right)=e_{child}$, and if so, it replaces the current edge assigned to $x_{child}$ with the edge $e_{newChild}$. If the replacement is successful, $e_{newChild}$ is added to the list of children of $e_{new}$ and $update(e_{child},e_{newChild})$ is called, because the edge $e_{child}$ has now become “obsolete” and its descendants must also be updated.

If the edge $e_{old}$ has no remaining children, the procedure checks whether edge $e_{new}$ has been “outdated” by another thread during the loop and, if so, it updates the children of $e_{new}$.

### 3.3. Rewire

The rewire procedure is used to replace the parent of vertex $x_{close}$ with vertex $x_{new}$ or one of its ancestors, provided that the cost of the resulting path is lower than that of the current path to $x_{close}$.

For each vertex $x_{current}$, which is either vertex $x_{new}$ or one of its ancestors up to a depth of $d_{max}$, the loop first checks whether vertices $x_{close}$ and $x_{current}$ can be properly connected, if so, it evaluates whether the cost of the path through $x_{current}$ is lower than the cost of the current path. If not, the loop exits and proceeds to the next vertex $x_{current}$. This procedure is repeated until the currently assigned to $x_{close}$ can be replaced with an edge leading to $x_{current}$ using the swapEdge procedure.

When the replacement is successful, the new edge is added to its parent’s list of children, the children of the “expired” edges are updated, and the old edge is removed from its parent’s list of children.

### 3.4. Fast Marching Methods

In this study, FMM was used to generate drone flight trajectories over extensive areas discretized into square grids. The implementation was based on the algorithm described in [15].

Similar to the RRT, the algorithm determines a path to the target that minimizes a function defined as the length of the flight path on which the UAV is within the radar recognition range.

**Figure 2:**
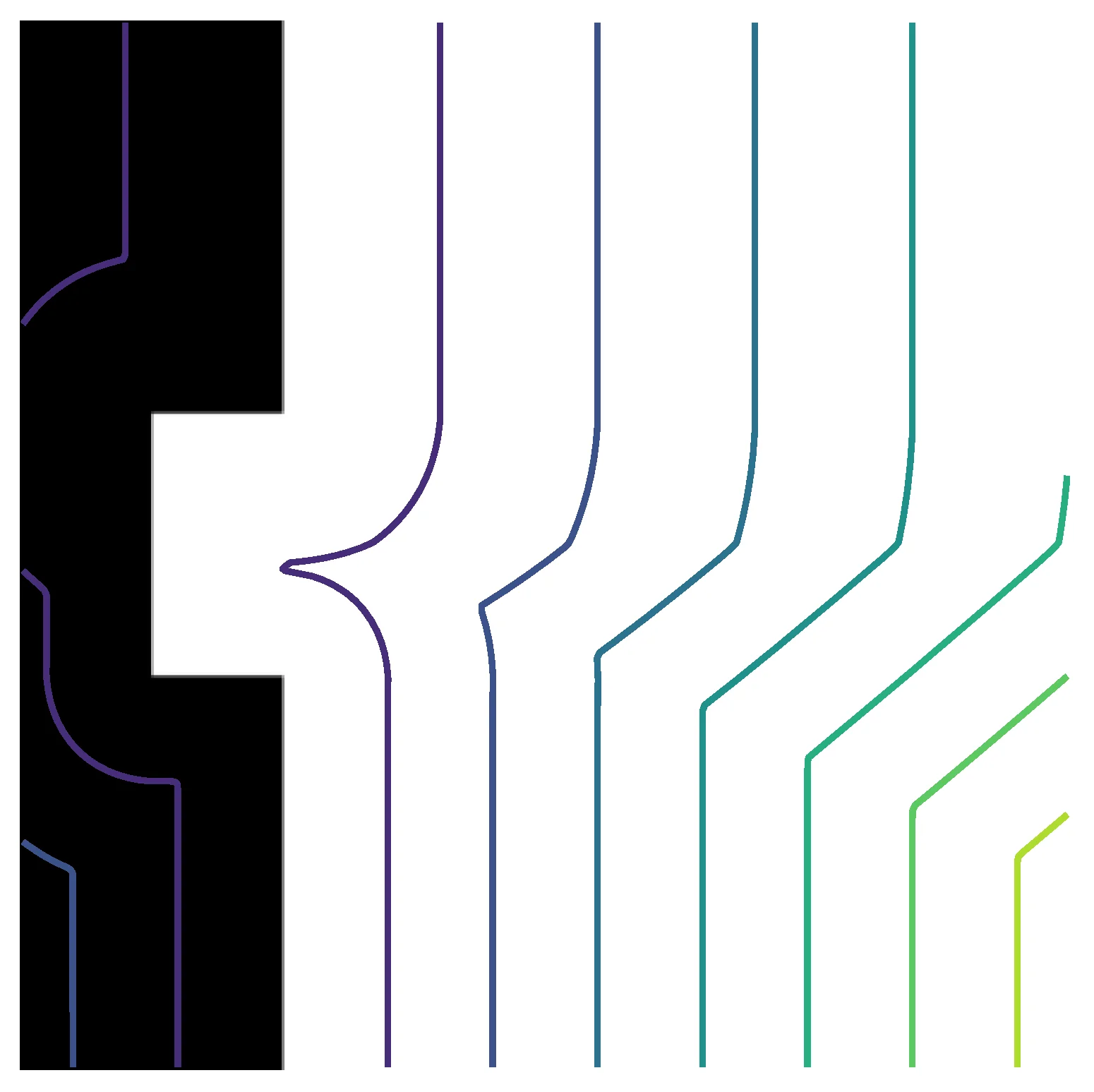
Visualization of the wavefront for the field f(x,y) = {1.5 for y>0 | 1 for y<0}. The set B, whose boundary represents the initial wavefront, is marked in black.

Sethian [22] described the FMM. It is an efficient numerical algorithm based on the concept of a wavefront propagating through a given medium. In such a medium, waves propagate forward at a specific speed that may vary with the medium’s properties. Consequently, when the medium changes, the waves may propagate non-concentrically due to variations in speed. A visualization of this algorithm is shown in Figure 2.

On a 2D map, the FMM method computes the time required for a wavefront to reach each point in space. The Eikonal equation describes the propagation of the wavefront (8) (see [23]), where *x* represents a point in space, f(x,y) denotes the expansion velocity, and T(x,y) is the time it takes for the wavefront to reach each point in space.

(8) ∇T(x,y)=1/f(x,y)

In this paper’s case study of radar field alignment verification, the probability of detecting a flying object depends on the wave propagation speed. The shortest time path is computed using gradient descent and corresponds to the trajectory with the lowest detection probability.

The following notations are used in Algorithm 5:

- B is a set of points within distance r=5 from $x_{start}$; *r* is also a radius of initial wavefront.
- F is a set of points labeled as *far*.
- A is a set of points labeled as *accepted*.
- C is a set of points labeled as *considered*.

**Algorithm 5** FMM Pathfinder**Require:** $x_{begin},x_{end},step\_size,loss\_landscape$ **Ensure:** path *p* from $x_{begin}$ to $x_{end}$
  1:B←−1;  2:$B^{\prime }\leftarrow +\infty$;  3:$F\leftarrow B^{\prime }$;  4:∂B←0;  5:A←∂B;  6:**for** $x_{i}\in F$ **do**  7:   $newVal\leftarrow EikonalUpdate(x_{i},loss\_landscape)$;  8:   **if** $newVal<x_{i}.value$ **then**  9:      $x_{i}.value\leftarrow newVal$10:   **end if**11:   $C\leftarrow x_{i}$12:**end for**13:**while** C not empty **do**14:  $x_{min}\leftarrow$ element of C with smallest x.value15:   $A\leftarrow x_{min}$16:   **for** each neighbor $x^{\prime }$ of $x_{min}$ **do**17:      **if** $x^{\prime }\in F$ **then**18:         $C\leftarrow x^{\prime }$19:      **end if**20:      **if** $x^{\prime }\notin A$ **then**21:         $newVal\leftarrow EikonalUpdate(x^{\prime },loss\_landscape)$22:         **if** $newVal<x^{\prime }.value$ **then**23:               $x^{\prime }.value\leftarrow newVal$24:         **end if**25:      **end if**26:   **end for**27:**end while**28:$path\leftarrow GradientDescent(x_{end},y)$

At the beginning of the algorithm’s execution, the values of T(x,y) are initialized for all map cells. Cells that belong to the initial wavefront ∂B are assigned a value of 0 and marked as *accepted*, while all remaining cells are assigned +∞ and flagged as F (*far*). Points inside B are inaccessible to the algorithm and are assigned the value of −1.

The next step is to compute a new value of T(x,y) for each cell in the set F using Algorithm 6, i.e.,  EikonalUpdate. If $T_{new}<T_{old}$, the cell’s label is changed from F (*far*) to C (*considered*).

Starting with the cell in C that has the smallest T(x,y), we set the flag to A (*Accepted*). Then, we compute the new value of T for all its neighbors that are not in A. If $T_{new}<T_{old}$, the cell’s value is updated to $T_{new}$. Each visited neighbor is assigned the flag C (*considered*), if it was previously F (*far*).

This process is repeated until the set C is empty.

After obtaining the time map from the algorithm described above, the optimal route is backtracked using Algorithm 7, i.e.,  GradientDescent. Starting from the endpoint, a gradient step of length a·Grad(x,y) (where Grad(x,y)=∇T(x,y)) is performed, and the resulting point is added to the *path* sequence. This procedure is repeated until the distance from the starting point is less than or equal to *r*.
**Algorithm 6** EikonalUpdate**Require:** *x*, *f* **Ensure:** arrival time T
  1:$T_{x}$← min$\left\{T\left(x_{i-1}\right),T\left(x_{i+1}\right)\right\}$  2:$T_{y}$← min$\left\{T\left(x_{i-1}\right),T\left(x_{i+1}\right)\right\}$  3:id←x.indices  4:**if** $|T_{x}-T_{y}|\leq 1/f_{id}$ **then**  5:   $T\leftarrow \frac{T_{x}+T_{y}}{2}+\frac{1}{2}\sqrt{{\left(T_{x}+T_{y}\right)}^{2}-2\left(T_{x}^{2}+T_{y}^{2}-\frac{1}{f_{id}}\right)}$  6:**else**  7:   $T\leftarrow min(T_{x}+1/f_{id},T_{y}+1/f_{id})$  8:**end if**  9:**return** T

**Algorithm 7** Gradient Descent**Require:** *x*, *T* **Ensure:** path *p*
  1:Grad←Gradient(T)  2:id←x.indices  3:**while** $x-x_{start}>r$ **do**  4:   $x\leftarrow x-a\cdot Grad_{id}$  5:   Append *x* to path  6:**end while**  7:**return** path

For trajectory planning purposes, Garrido et al. [11] introduced a modified FMM called FM^2^. Its operation is based on applying the FMM algorithm twice. The FMM algorithm is limited to finding paths only within the scalar field f(x,y) and does not account for binary obstacles, which represent “forbidden zones”. This limitation arises because the algorithm seeks the globally shortest path, and therefore tends to move as close as possible to obstacles while avoiding them.

The FM^2^ algorithm addresses this problem as follows:

1. In the first FMM execution, the initial wavefront is defined as the boundary of the obstacle to be avoided. This produces the obstacle distance field.
2. In the second FMM iteration, the algorithm is initialized from the starting point, using the field f(x,y) obtained in step 1. In this way, the algorithm balances the path length with the distance from the obstacle.

### 3.5. Other Methods

In many practical optimization problems, the goal is to minimize the objective function (also called the fitness or cost function). Often, the structure or nature of the device or phenomenon being studied is not provided as a mathematical model.

A black-box scenario in which the function values of the evaluated search points are the only available information, and gradients are not available.

Applications include, among others: aerodynamics (e.g., shape optimization), economics (e.g., portfolio selection), or operations research.

Several optimization techniques have been developed for trajectory calculation, the most popular of which are swarm-based algorithms (e.g., Particle Swarm Optimization (PSO)) or evolutionary algorithms (e.g., CMA-ES, Genetic Algorithm (GA)). GAs come in a wide variety, differing in the methods of mutation, initial population generation, and so on. Selected examples are described by Ragusa et al. [24].

In recent years, CMA-ES has proven effective for continuous optimization of problems with unknown mathematical models. The effectiveness of CMA-ES has been demonstrated across benchmarks and various practical applications. This paper uses an available Python v.3.15 implementation of the CMA-ES algorithm developed by Nomura et al. [25,26]. The algorithm implementation incorporates key features of CMA-ES, including an adaptive learning rate that adapts to various scenarios.

## 4. Results

In this paper, the trajectory generation speed was analyzed using the RRT, FMM, and CMA-ES algorithms for radar coverage areas of 1400 × 1500 grids (in terrain more than 100 × 100 km). The area was divided into equal-sized squares, and radar coverage was discretized within these regions.

The radar-coverage maps used in the numerical experiments were generated using radar-specific computational models corresponding to selected medium-range radar systems. The maps represent the estimated detection probabilities for assumed target radar cross sections and flight altitudes. Independent measurement-based validation of these coverage maps is not presented in this study.

Testing environment specifications:

- Intel^®^ i7-9700^®^ central processing unit (CPU) @ 3.0 GHz (8 cores and 8 threads; 6 cores used in calculations) (Intel Corporation, Santa Clara, CA, USA),
- 32.0 GB random-access memory (RAM),
- 512 GB solid-state drive (SSD) storage,
- 64-bit Windows 10 operating system (OS).

Each test scenario was executed independently 20 times, using different random seeds for the stochastic algorithms. The execution times reported in Table 1 represent the mean values and standard deviations calculated across these runs. The PQRRT* implementation used six computational threads. The reported execution times exclude the time required for initializing algorithm-specific data structures and generating radar-coverage maps.

The objective of the test is to identify UAV trajectories associated with the lowest cumulative detection probability for a given radar deployment.

Trajectories associated with delayed entry into the radar coverage area are particularly relevant for identifying potential coverage gaps. If the resulting trajectories indicate insufficient protection of the monitored area, the analyst can manually adjust the radar placement and re-determine the flight trajectories using the PQRRT*, FMM, and CMA-ES algorithms.

**Figure 3:**
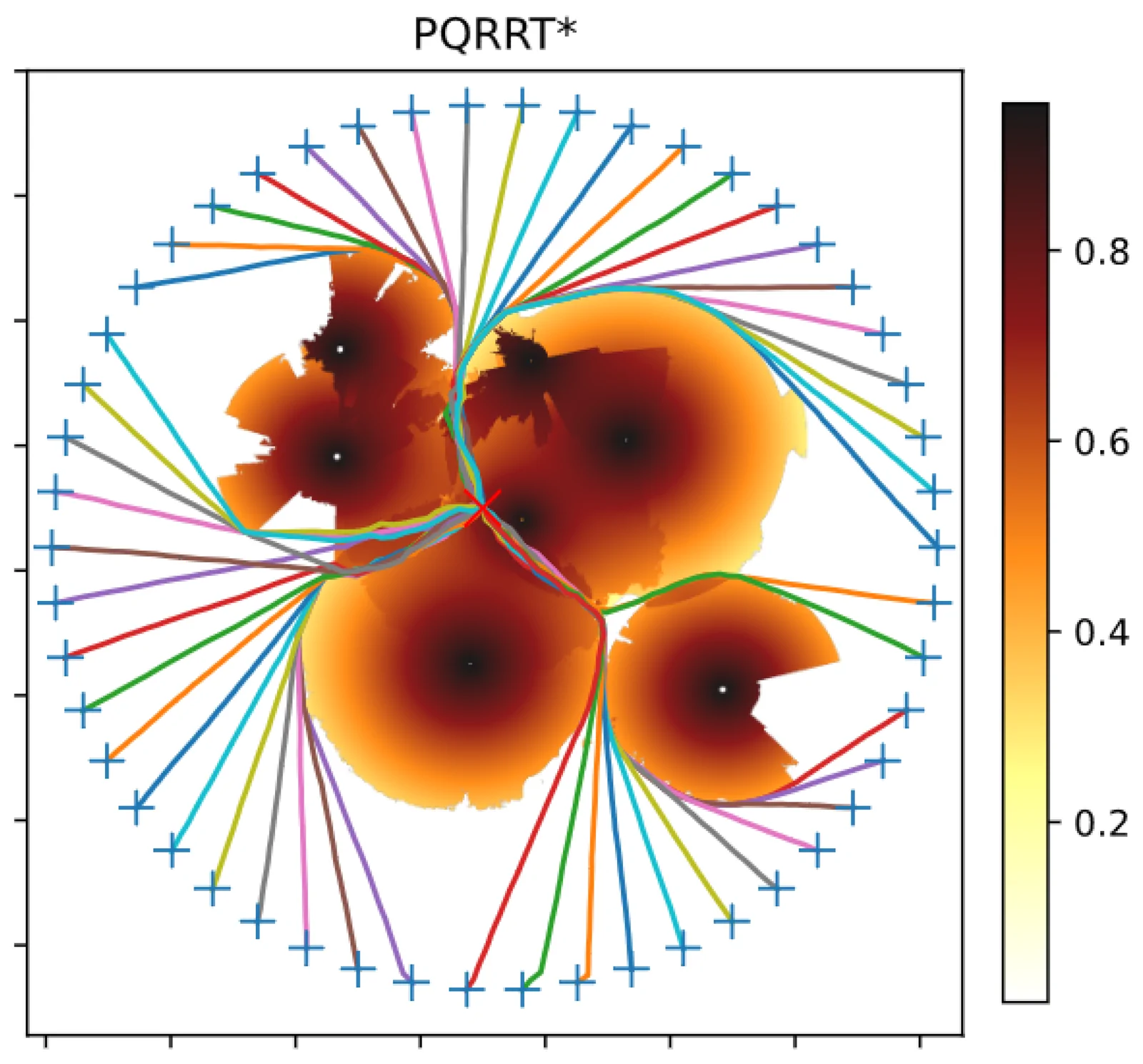
Trajectory generated using the PQRRT* algorithm.

Figure 3 illustrates the results of generating several trajectories for one of the test radar coverage scenarios. Six radars were deployed within the area, and their coverage zones are shown as partial circles. Darker colors indicate regions with stronger radar fields, corresponding to a higher probability of detecting an aircraft of a given RCS at a specific altitude. Conversely, lighter colors indicate weaker radar fields, with lower detection probability. The target is marked with a red cross. It can be observed that the individual trajectories were planned to avoid areas with high field strength. The trajectories intersect the boundaries between radar coverage regions in certain segments.

**Figure 4:**
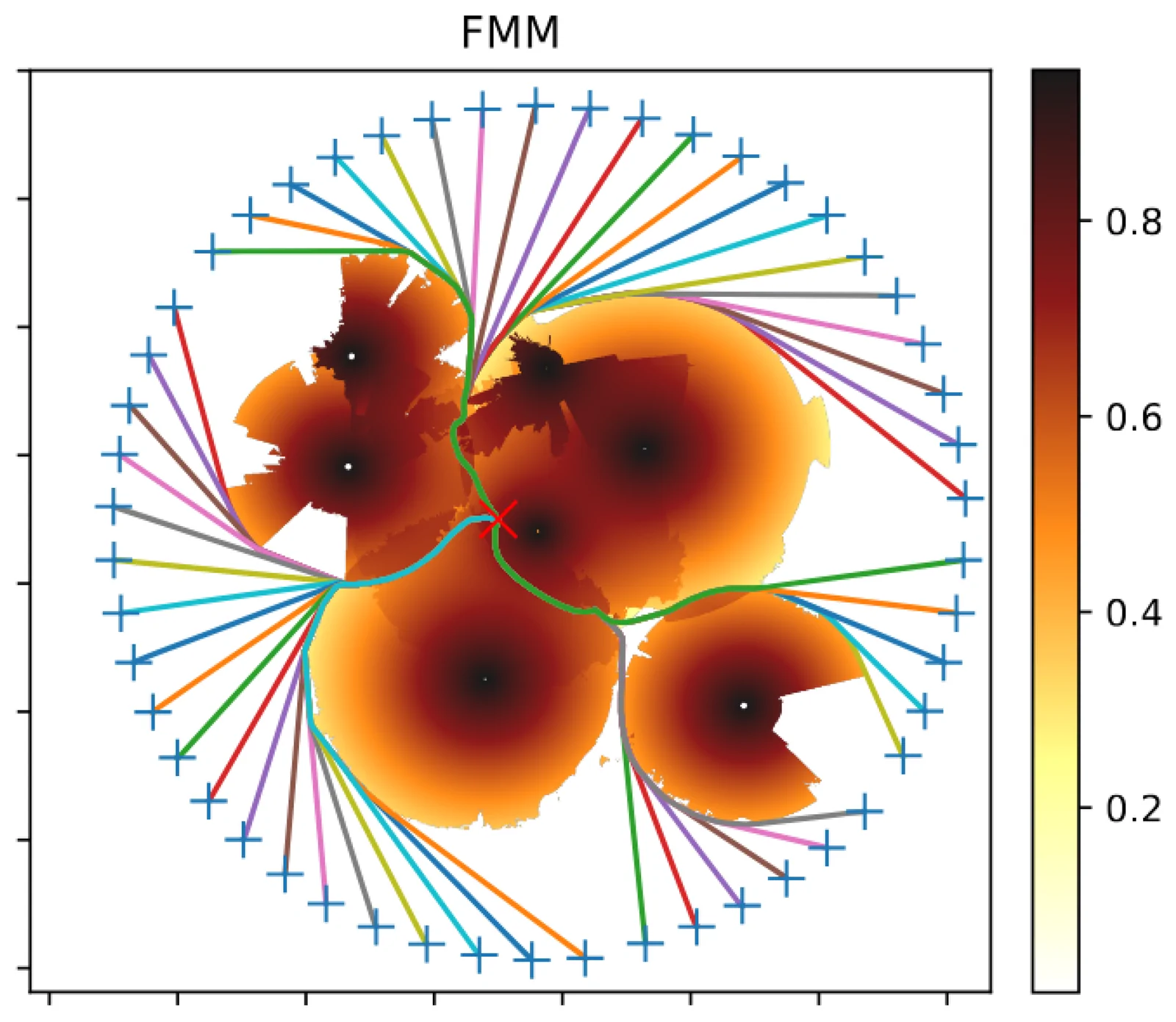
Trajectory generated using the FMM algorithm.

Figure 4 presents the results of trajectory calculation for the same area using the FMM algorithm. The same number of radars were deployed in the identical locations. The individual trajectories were generated to avoid areas with high field strength. The results of both algorithms are similar and comparable.

The performance of the CMA-ES evolutionary algorithm was also evaluated, yielding results comparable to those obtained with the other algorithms in terms of detection probability. However, in the considered large-scale grid-based scenarios, CMA-ES is less computationally efficient than the specialized trajectory-planning algorithms evaluated in this study. Nevertheless, CMA-ES is included as a general-purpose optimization baseline to illustrate the computational differences between evolutionary optimization and specialized trajectory-planning methods.

**Figure 5:**
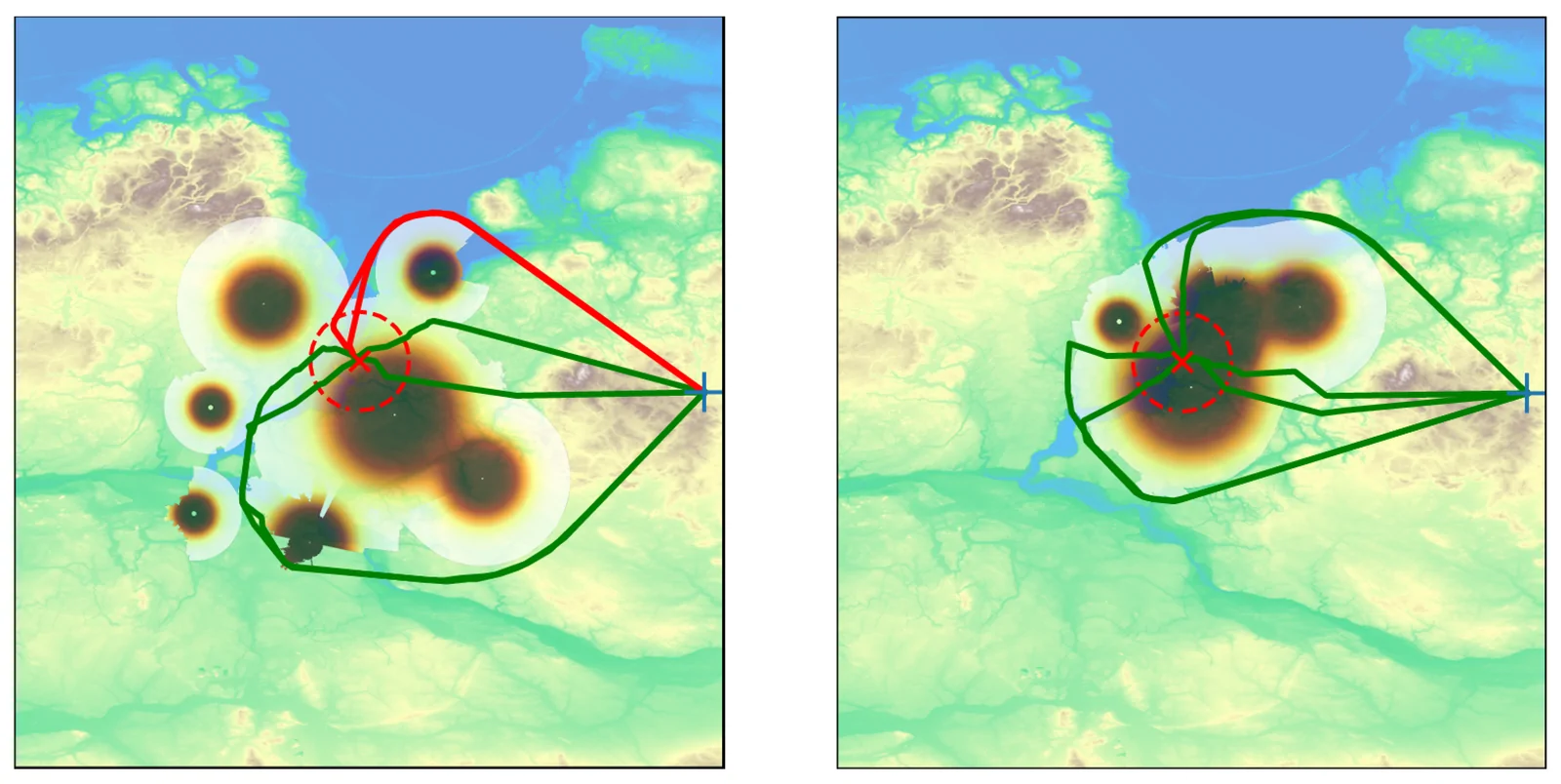
Flight trajectories of several UAVs calculated using the PQRRT* algorithm for a selected initial (**left figure**) and manually adjusted (**right figure**) radar position in the field. Colors in the figure represent the probability of detecting a flying object with a given RCS moving at a given altitude. The highest probability of detecting a UAV is in the dark brown area.

**Figure 6:**
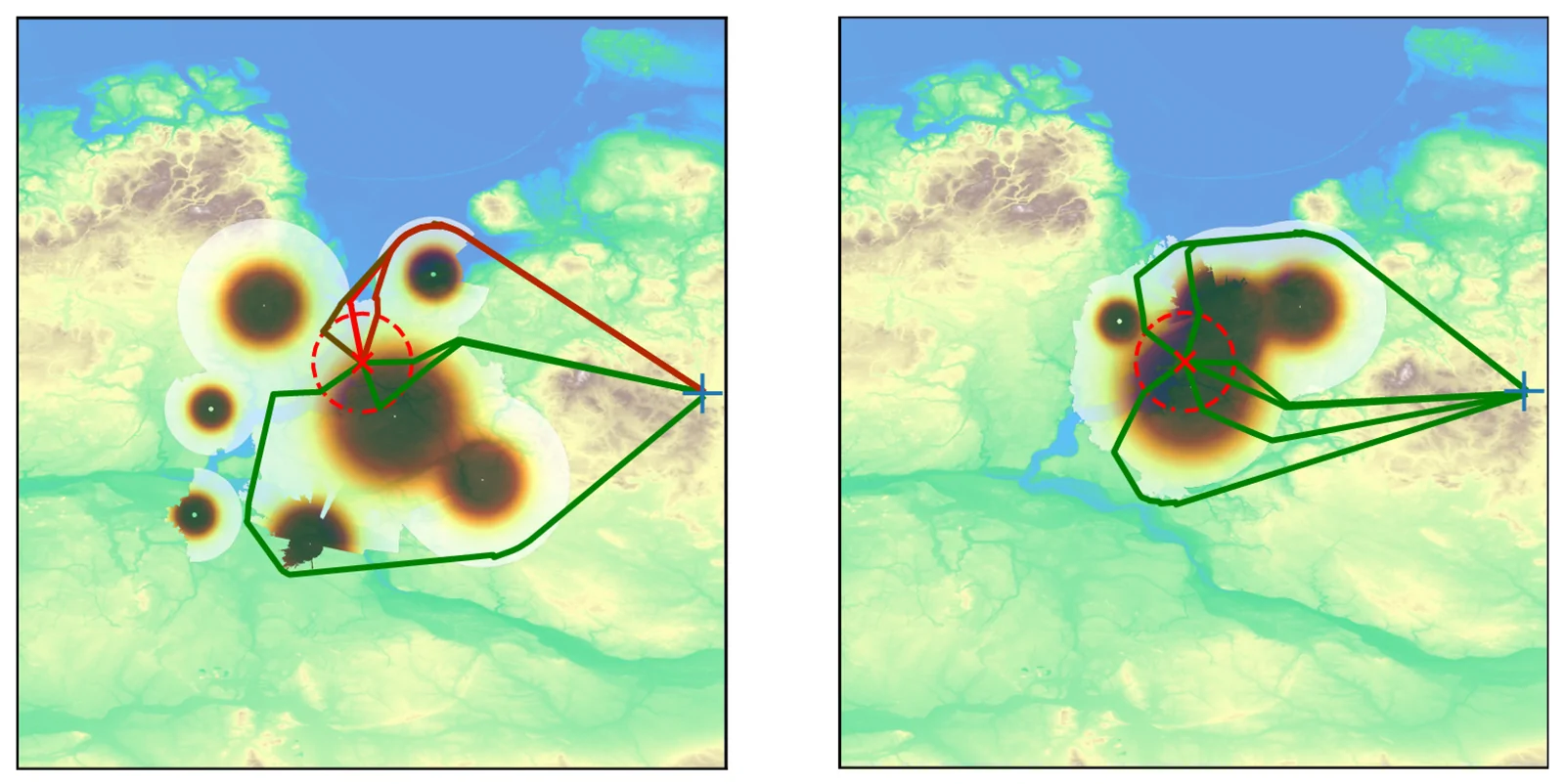
Trajectories determined using the FMM algorithm. FMM tends to determine UAV flight trajectories as close together as possible, which is not always advantageous. The probability of detecting a flying object is highest near the radar and decreases with distance, especially when radar signals are obscured by uneven terrain.

**Figure 7:**
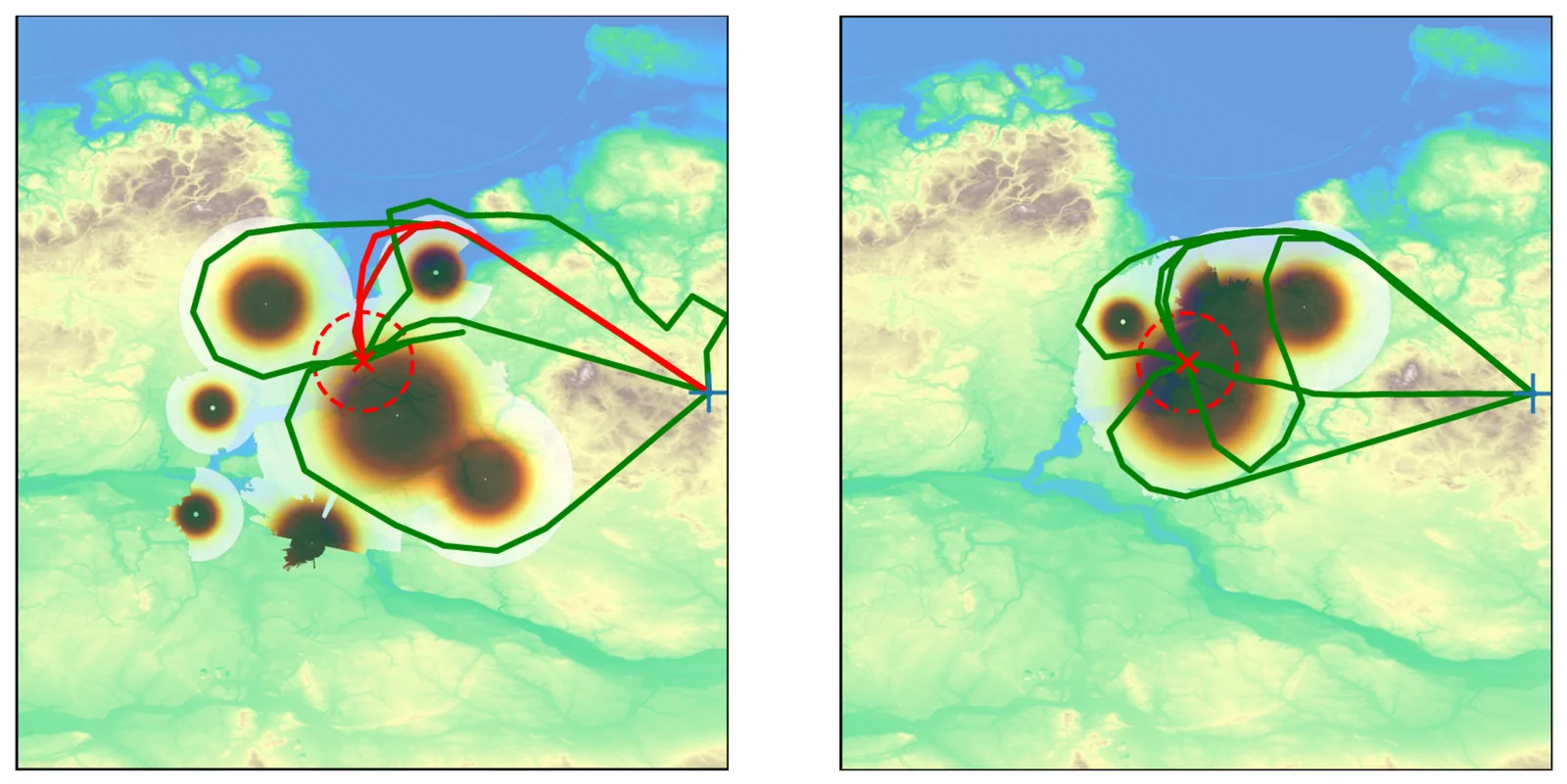
Flight trajectories of several unmanned aerial vehicles calculated using the CMA-ES algorithm for a selected initial (**left figure**) and manually corrected (**right figure**) radar position in the field. The CMA-ES algorithm tends to generate paths with lengths significantly different from those generated by the other algorithms.

Figure 5, Figure 6 and Figure 7 present the results of generating six trajectories using the FMM, PQRRT*, and CMA-ES algorithms. Several radars were deployed within the area at locations predefined by the analyst. The individual trajectories indicate potential drone entry paths into radar-protected zones. It was assumed that the UAVs depart from a common initial region, while six distinct trajectories approaching the radar-covered area from different directions were generated. This configuration enables the radar deployment to be evaluated against multiple representative approach directions and facilitates the identification of potential coverage gaps. This approach enables the trajectories to separate near the initial region, after which the UAVs follow paths selected to minimize the time spent within radar coverage.

These figures also show the results of calculating six trajectories using the FMM, PQRRT*, and CMA-ES algorithms after adjusting the radar positions in the field. The method of correcting radar placement is not the subject of this work. We assume it is performed manually.

As in the previous case, it was assumed that the UAVs departed from a common initial region, and six distinct trajectories representing different approach directions were generated. For the corrected radar deployment, the resulting trajectories indicate that the potential coverage gaps were substantially reduced.

Table 1 presents the trajectory calculation times for each algorithm in areas divided into 1-km squares. Tests were conducted for selected radar types in regions with side lengths exceeding 100 km, resulting in square meshes composed of tens of thousands of elements.

The results in Table 1 confirm the effectiveness of the FMM and PQRRT* algorithms and highlight their advantages over the CMA-ES evolutionary algorithm. For calculations involving real-world scenarios (such as determining trajectories over a large area), CMA-ES exhibits substantially higher computational costs under the considered experimental conditions. The parallel version of the RRT algorithm, which uses non-blocking structures (without synchronization mechanisms or access to shared areas), demonstrates efficiency comparable to that of FMM. Moreover, it is more readily adaptable for incorporating UAV flight dynamics.

Table 2 shows the probabilities of detecting airborne platforms for two radar location scenarios: initial and corrected. The initial radar configuration exhibits coverage gaps that allow some aerial objects to approach the monitored area with relatively low probabilities of detection. After manually correcting the radar location, airborne platforms are detected with a higher probability, regardless of the algorithm used to generate the flight trajectories. The PQRRT* algorithm found the lowest-cost trajectories; however, for the considered radar deployment, these trajectories were still associated with a high probability of detection.

It should be emphasized, however, that the presented method does not guarantee the protection of a target in every case. The results are primarily influenced by the number of available radars and the characteristics of the terrain. In complex terrain, a limited number of radars may result in insufficient coverage of certain regions.

## 5. Discussion and Summary

Supporting radar deployment assessment for the effective monitoring of designated areas requires methods capable of identifying potential UAV trajectories associated with reduced radar coverage.

The trajectory-planning algorithms presented in this paper can be used for this purpose. For preliminary radar-deployment assessment, the proposed framework focuses on identifying representative low-detection-probability UAV trajectories without explicitly incorporating UAV kinematic and dynamic constraints. This simplification facilitates computationally efficient trajectory generation but limits the direct applicability of the resulting paths to real UAV flight operations.

The adopted detection-probability field is assumed to be time-independent and does not explicitly account for UAV speed, the time spent within individual grid cells, or radar scan rates. Furthermore, the generated piecewise-linear trajectories may not satisfy platform-specific constraints, such as minimum turning radius or climb and descent limits. Incorporating such constraints or applying trajectory-smoothing techniques could modify the resulting paths and their accumulated detection probabilities. A quantitative assessment of these effects is left for future research.

The radar-coverage model adopted in this study provides the spatial detection-probability field required by the trajectory-planning algorithms. The model incorporates several simplifying assumptions, including the neglect of multipath fading at low altitudes, surface clutter, and variations in RCS of UAV due to changes in aspect angle. However, the proposed framework is not limited to this particular coverage model, and more advanced propagation models, including those based on the Parabolic Equation Method (PEM) [27], could be incorporated to generate higher-fidelity coverage maps. Evaluating the impact of alternative, more accurate propagation models on trajectory-planning results, however, remains beyond the scope of the present study.

The presented algorithms, particularly PQRRT*, enable efficient determination of UAV trajectories in large-scale radar-covered areas represented by 2D spatial grids. The numerical experiments conducted in this study consider detection-probability fields at specified flight altitudes. Although the underlying planning approach may be extended to three-dimensional environments, its computational performance and practical applicability in 3D scenarios have not been evaluated in the present study.

As the threat posed by UAVs grows, new opportunities for threat identification using synthetic aperture radar (SAR) systems are emerging. Falecki et al. [28] conducted tests as part of the Passive Radar Measurements with Space Transmitters of Opportunity (PaRMeSTO) trials, carried out by the SET-320-RTG research task group (RTG), operating within the North Atlantic Treaty Organization (NATO) Science and Technology Organization (STO). The experiments demonstrated the feasibility of detecting unmanned aerial vehicles and generating images of them using bistatic SAR with ground-based receivers. The validity of this approach was confirmed, highlighting its potential for enhancing the protection of borders and critical infrastructure. Consequently, the challenge of predicting the attack trajectories of drones is becoming increasingly complex.

Further research will focus on two main issues. First, improving the efficiency of 3D flight trajectory determination. Second, assessing the feasibility of implementing coordinated routes for multiple drones flying at similar altitudes in areas covered by radar. In this context, the algorithms for assessing and prioritizing threats (as presented in the work by Krenc et al. [29]) will be utilized.

## Figures and Tables

**Table 1:** The times required to determine a specific number of trajectories in the test environment with radar coverage for each algorithm are presented. For the PQRRT* algorithm, the WPTNo (max. no. of waypoints) parameter specifies the maximum number of RRT tree nodes that can be generated for a given maximum number of iterations. In the FMM algorithm, the stepSize parameter denotes the gradient step length, which determines the number of vertices along the broken-line path we move in the so-called “time field”. The FMM generates this field and indicates the time required for a wave to travel from the source to a specific point in space. In the CMA-ES algorithm, the nWaypoints parameter specifies the number of vertices along the route, which is fixed and predetermined.

Test No.	Test Env. Size	No. Traj. Searched	Algorithm	Parameters WPTNo(PQRRT*) stepSize(FMM) nWaypoints(CMA-ES)	Standard Deviation [s]	Average Time [s]
1	1400×1500	1	PQRRT*	$2\times 10^{5}$	0.03	0.7
2	1400×1500	6	PQRRT*	$2\times 10^{5}$	0.03	4.0
3	1400×1500	8	PQRRT*	$2\times 10^{5}$	0.03	5.3
4	1400×1500	1	PQRRT*	$3\times 10^{5}$	0.01	1.4
5	1400×1500	6	PQRRT*	$3\times 10^{5}$	0.03	9.8
6	1400×1500	8	PQRRT*	$3\times 10^{5}$	0.08	15.0
7	1600×1900	1	PQRRT*	$3\times 10^{5}$	0.02	2.6
8	1600×1900	6	PQRRT*	$3\times 10^{5}$	0.03	11.6
9	1600×1900	8	PQRRT*	$3\times 10^{5}$	0.04	18.7
10	1800×2000	1	PQRRT*	$3\times 10^{5}$	0.02	4.1
11	1800×2000	6	PQRRT*	$3\times 10^{5}$	0.03	13.8
12	1800×2000	8	PQRRT*	$3\times 10^{5}$	0.05	21.4
13	2000×2400	1	PQRRT*	$3\times 10^{5}$	0.03	5.7
14	2000×2400	6	PQRRT*	$3\times 10^{5}$	0.04	16.2
15	2000×2400	8	PQRRT*	$3\times 10^{5}$	0.06	25.8
16	1400×1500	1	FMM	100	0.01	1.6
17	1400×1500	1	FMM	$10^{3}$	0.01	1.2
18	1400×1500	1	FMM	$10^{4}$	0.01	1.1
19	1400×1500	8	FMM	100	0.04	12.9
20	1400×1500	8	FMM	$10^{3}$	0.03	9.5
21	1400×1500	8	FMM	$10^{4}$	0.03	8.5
22	1400×1500	1	CMA-ES	15/1500	0.03	4.3
23	1400×1500	1	CMA-ES	50/15.000	–	>120
25	1400×1500	8	CMA-ES	15/1500	0.09	34.3
26	1400×1500	8	CMA-ES	50/15.000	–	>1440

**Table 2:** Probabilities of detecting incoming air targets for each algorithm before and after radar position modification. Probabilities are listed in ascending order. For the evaluated radar configurations and generated trajectories, the manual adjustment of radar positions resulted in higher detection probabilities, indicating a reduction in potential coverage gaps within the considered scenarios. Detection probabilities are sorted independently in ascending order for each algorithm and radar configuration. Therefore, trajectory numbers indicate positions in the sorted results and do not represent corresponding trajectories across different algorithms.

Test	Detect.	Detect.	Detect.	Detect.	Detect.	Detect.
	Prob.	Prob.	Prob.	Prob.	Prob.	Prob.
	Traj. 1	Traj. 2	Traj. 3	Traj. 4	Traj. 5	Traj. 6
PQRRT* initial	0.124	0.165	0.975	0.999	0.999	1.0
PQRRT* final	0.958	0.999	0.999	1.0	1.0	1.0
FMM initial	0.325	0.445	0.592	0.946	0.999	1.0
FMM final	0.999	1.0	1.0	1.0	1.0	1.0
CMA-ES initial	0.27	0.37	0.936	0.998	0.999	1.0
CMA-ES final	0.980	0.988	0.999	1.0	1.0	1.0

## Data Availability

Digital maps data is available publicly in Internet. Selected algorithms can be made available upon request.
